# Innovative methods for health monitoring in Europe: results of a cross-sectional study: Brigid Unim

**DOI:** 10.1093/eurpub/ckac131.562

**Published:** 2022-10-25

**Authors:** B Unim, MJ Forjaz, M Thissen, N Schutte, L Palmieri

**Affiliations:** 1 Cardiovascular, Endocrine-metabolic Diseases, National Institute of Health, Rome, Italy; 2 National Center of Epidemiology, Health Institute Carlos III, Madrid, Spain; 3 Department for Epidemiology and Health Monitoring, Robert Koch Institute, Berlin, Germany; 4 Epidemiology and Public Health, Sciensano, Brussels, Belgium

## Abstract

**Background:**

Innovative solutions are used to monitor the spread of COVID-19, to research and develop vaccines, and to ensure online privacy and security. The aim of the study is to investigate which innovative methods, including algorithms and digital tools (e.g., social media, artificial intelligence, contact tracing applications) are used to monitor health issues related to COVID-19 in Europe, and who is using them.

**Methods:**

A questionnaire was developed and administered online to European countries’ representatives and stakeholders of the project Population Health Information Research Infrastructure (PHIRI). The survey investigated the use of innovative solutions and digital tools in Europe to monitor COVID-19 and vaccination programs, to research and develop diagnostics and teleconsultations, and to fight online disinformation. Legislative and ethical aspects were also considered. A descriptive data analysis was performed.

**Results:**

19 responses were collected from 14 countries. Digital tools are used to monitor COVID-19 (13/14 countries), vaccination programs (12/14), for telemedicine (7/14), and to fight disinformation (10/14). Specific algorithms to detect the patterns of the pandemic spread are available in five countries. The main target groups of the tools are the general population, healthcare providers, patients and epidemiologists. The uptake rate of the tools ranged 5-100% across countries. Measures to evaluate the impact of digital tools (e.g., user surveys, reviews, evaluation teams) have been adopted in seven countries. Information on legislative and ethical aspects related to the use of digital solutions are available in 10 countries.

**Conclusions:**

The development and use of innovative methods for population health monitoring and research purposes have been the key to mitigate the COVID-19 pandemic. Improving the uptake rate, impact assessment of digital tools and fight against disinformation could enhance countries’ preparedness for future pandemics.

**Key messages:**

• Given that digital solutions are deployed in population health monitoring, research, and for online privacy and security, they have a key role in mitigating the COVID-19 pandemic.

• Enhancement of the uptake rate and assessment of digital tools, and fight against disinformation could strengthen countries’ preparedness for future pandemics.

